# Predictors of Outcomes in Patients with EGFR-Mutated Non-Small Cell Lung Cancer Receiving EGFR Tyrosine Kinase Inhibitors: A Systematic Review and Meta-Analysis

**DOI:** 10.3390/cancers11091259

**Published:** 2019-08-28

**Authors:** Carlo Buonerba, Simona Iaccarino, Pasquale Dolce, Martina Pagliuca, Michela Izzo, Luca Scafuri, Ferdinando Costabile, Vittorio Riccio, Dario Ribera, Brigitta Mucci, Simone Carrano, Fernanda Picozzi, Davide Bosso, Luigi Formisano, Roberto Bianco, Sabino De Placido, Giuseppe Di Lorenzo

**Affiliations:** 1 Regional Reference Center for Rare Tumors, Department of Oncology and Hematology, AOU Federico II of Naples, 80131 Naples, Italy; 2 National Reference Center for Environmental Health, Zoo-prophylactic Institute of Southern Italy, 80055 Portici, Italy; 3 Department of Clinical Medicine and Surgery, University Federico II of Naples, Via Pansini 5, 80131 Naples, Italy; 4 Department of Public Health, Federico II University of Naples, 80131 Naples, Italy; 5 Department of Medicine and Health Sciences ‘Vincenzo Tiberio’, University of Molise, 86100 Campobasso, Italy

**Keywords:** non-small cell lung cancer, epidermal growth factor receptor tyrosine kinase inhibitors, sex

## Abstract

Some commonly available patient or disease characteristics may be associated with progression-free survival (PFS) and overall survival (OS) in EGFR-mutant non-small cell lung cancer (NSCLC) patients receiving EGFR-TKIs (epidermal growth factor receptor - tyrosine kinase inhibitors). We performed a systematic review and meta-analysis of randomized control trials (RCTs) to explore differences in outcomes associated with EGFR-TKIs among subgroups of EGFR-mutant NSCLC patients. Pooled HRs for progression or death (PFS-HRs) and pooled HRs for death (OS-HRs) were compared among sub-groups defined according to baseline clinical and demographic variables as well as type of EGFR mutation. In the entire assessable population of 4465 EGFR-mutant NSCLC patients, significant interactions with PFS were found for gender (males vs. females; pooled ratio of the PFS-HRs = 1.2; 95% CI 1.12–1.56), smoking history (smokers vs. non-smokers; pooled ratio of the PFS-HRs = 1.26; 95% CI 1.05–1.51), and type of EGFR mutation (patients with exon 21 L858R mutation vs. exon 19 deletion; pooled ratio of the PFS-HRs = 1.39; 95% CI 1.18–1.63). Male patients, smokers and patients with EGFR exon 21 L858R mutation may derive less benefit from EGFR-TKIs compared to female patients, non-smokers and patients with EGFR exon 19 deletion.

## 1. Introduction

Lung cancer is responsible for 11.6% of the 18.1 million new cancer cases and for 18.4% of the 9.6 million cancer deaths estimated in 2018 [1]. While chemotherapy is unable to extend median overall survival beyond a year in unselected patients with advanced non-small cell lung cancer (NSCLC) [2], major improvements in prognosis have been achieved with immunotherapy and targeted therapy in selected patient populations [3]. 

The epidermal growth factor receptor (EGFR) is the main actionable target in NSCLC, with up to 50% of patients presenting activating EGFR mutations [4]. Three generations of epidermal growth factor tyrosine kinase inhibitors (EGFR-TKIs) are currently available in clinical practice, with overall improved outcomes in terms of efficacy and safety vs. standard chemotherapy in EGFR-mutant NSCLC patients. While first-generation agents (erlotinib, gefitinib, icotinib) reversibly bind to EGFR, second-generation agents (afatinib, dacomitinib) bind to both EGFR and HER2 (human epidermal growth factor receptor 2) irreversibly. Resistance to first- and second-generation EGFR inhibitors is frequently associated with the selection of T790M EGFR mutant clones, which are sensitive to the third-generation agent osimertinib [5]. 

Some common patient or disease characteristics, such as gender [6], performance status [6], ethnicity [7], brain metastasis [8], and type of EGFR mutation [9] may have an independent prognostic value in EGFR-mutant NSCLC, but some of these variables may be also predictive of EGFR-TKI efficacy, as shown in published meta-analyses of randomized control trials (RCTs) [10,11,12]. Nevertheless, rigorous quantitative analysis of differences in efficacy outcomes according to clinical subgroups based on an updated systematic review of randomized control trials of first-, second-, and third-generation EGFR-TKIs has not been yet performed, to the best of our knowledge.

In this work, we performed a comprehensive systematic review and meta-analysis of RCTs of EGFR-TKIs vs. other treatments as well as those comparing third- and second-generation vs. first-generation EGFR-TKI. The meta-analytic approach reported by Fisher [13] et al. was followed to explore the effect of common patient or disease characteristics as predictor variables of efficacy outcomes in NSCLC patients harboring EGFR mutations.

## 2. Results

### 2.1. Eligible Articles

**Figure 1:**
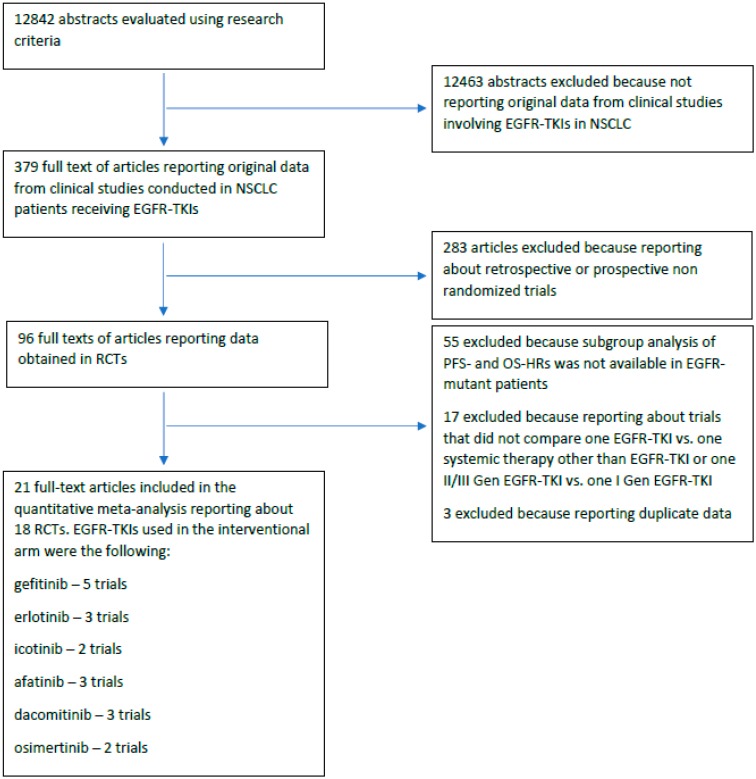
Flow-diagram of the systematic review. NSCLC = non-small cell lung cancer; Gen = generation; EGFR-TKIs = epidermal growth factor tyrosine kinase inhibitors; RCTs = randomized controlled trials; PFS = progression-free survival; OS = overall survival; HR = hazard ratio.

Our database search retrieved 12,842 abstracts. Of the 379 full-text clinical studies involving EGFR-TKIs in NSCLC that were evaluated, 96 full-text articles reporting data from randomized control trials were identified and 21 articles were finally included in the quantitative meta-analysis (Figure 1 and Table 1) [14,15,16,17,18,19,20,21,22,23,24,25,26,27,28,29,30,31,32,33,34].

Thirteen articles included reported about RCTs in EGFR-mutant NSCLC testing one first generation EGFR-TKIs in the interventional arm. One article reported PFS data in patients receiving chemotherapy plus gefitinib vs. chemotherapy after progression on first-line gefitinib(IMPRESS) [14]; one article reported PFS and OS data obtained in patients treated with first-line gefitinib vs. chemotherapy (WJTOG3405) [15]; one article reported PFS data obtained in the sub-group of EGFR-mutant NSCLC patients enrolled in the IPASS trial of fist-line gefitinib vs. carboplatin/paclitaxel [16]; two articles reported DFS data obtained with gefitinib vs. chemotherapy [17] and with gefitinib plus chemotherapy vs. chemotherapy [18], respectively, administered as adjuvant therapy after surgical resection; two articles reported PFS and OS data obtained in patients treated with first-line erlotinib vs. chemotherapy enrolled in the ENSURE [19] and EURTAC trials [20], respectively; three articles reported data obtained in the OPTIMAL-CTONG-0802 trial, of which two articles reported PFS data [21,22], and one reported OS data [23]; one article reported PFS and OS data obtained in patients randomized to first-line icotinib vs. chemotherapy enrolled in the CONVINCE trial [24]; and one article reported PFS and OS data in the BRAIN trial enrolling patients with brain metastases randomized to icotinib vs. whole brain irradiation plus chemotherapy [25]. 

Of the articles included that reported about clinical trials testing one of the second-generation EGFR-TKIs in the interventional arm, two articles reported about PFS data obtained in EGFR-mutant NSCLC patients randomized to afatinib vs. chemotherapy in a first-line setting in the LUX3 [26] and LUX6 [27] trials, of which updated OS data were provided by Yang et al. in a separate article [29]; one article (LUX Lung 7) [28] reported PFS data about afatinib vs. gefitinib as first-line treatment of EGFR-mutant NSCLC; one article reported PFS data obtained in EGFR-mutant patients randomized to dacomitinib vs. gefitinib in a first-line setting in the ARCHER 1050 trial [30], while updated OS data were reported in a separate article [31]; finally, one article was included because it provided pooled PFS and OS data obtained in the subgroup of EGFR mutant patients enrolled in two similarly designed randomized control trials of dacomitinib vs. erlotinib as second- or third-line therapy (ARCHER 1009 and A7471028) [32].

Of the articles included that reported data about RCTs testing one of the third-generation EGFR-TKIs in the interventional arm, one article reported PFS data obtained in patients with EGFR T790M mutation progressing after first-line EGFR-TKI therapy and randomized to osimertinib or chemotherapy [33]; another article reported PFS data obtained in EGFR-mutant patients randomized to osimertinib or a first-generation EGFR-TKI [34]. Overall, the 21 articles included reported PFS and/or OS data obtained in 18 randomized controlled trials. Of these, 3 were randomized phase II trials and 15 were phase III trials, while 3 were placebo-controlled and 15 were open label. Two trials were conducted in the adjuvant setting, 12 were conducted in the first-line setting and four in second- and/or later-line settings. Open label trials had a Jadad score of 3, while the 3 placebo-controlled trials had a Jadad score of 5.

### 2.2. Quantitative Synthesis

**Figure 2:**
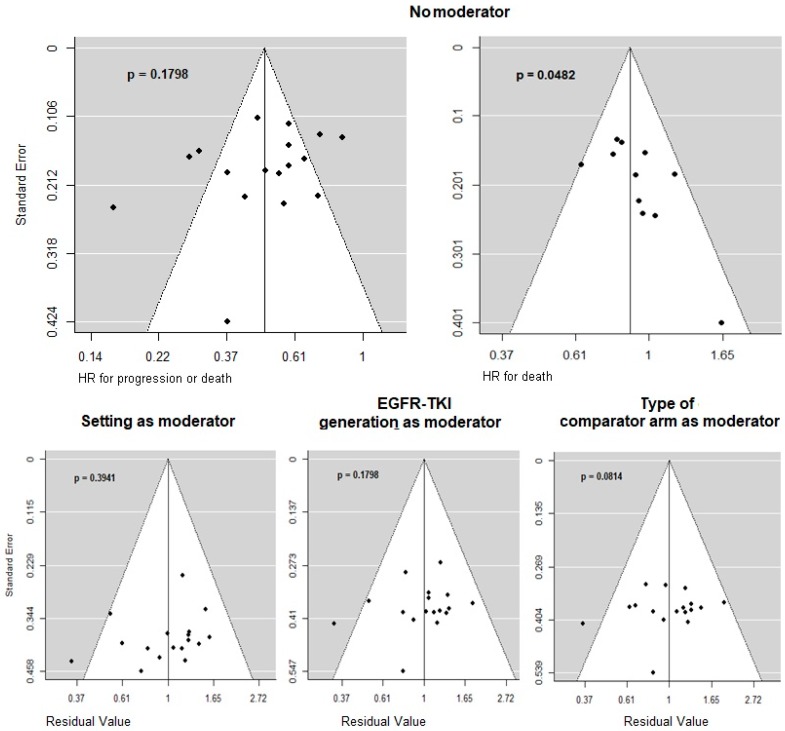
Funnel plots for OS- and PFS-HR. Reporting bias was assessed by visually evaluating asymmetry and by statistical test, for which *p*-values are reported. The y-axis reports standard error in all graphs. Above, the x-axis reports the effect sizes (HR for progression or death on the left and the HR for death on the right). Below, the x-axis reports the residual values instead of effect sizes considering setting, EGFR-TKI generation, and type of comparator arm as moderator variables.

Pooled data from a total of 4465 assessable EGFR-mutant NSCLC patients were included in the quantitative synthesis. Hazard ratios for progression or death (PFS-HRs) in EGFR-mutant patients were obtained from all the 18 trials included, while hazard ratios for death (OS-HRs) were available for 12 trials. The HRs of the ARCHER 1009 and A7471028 trials were analyzed as pooled HRs computed on individual-level patient data of these two trials. The pooled PFS-HR was 0.48 (95% CI 0.40–0.59), while the pooled OS-HR was 0.88 (95% CI 0.79–0.98). Regarding PFS-HR, no reporting bias or asymmetry was evident. Regarding OS-HR, funnel plot asymmetry appeared to be significant (Figure 2).

**Figure 3:**
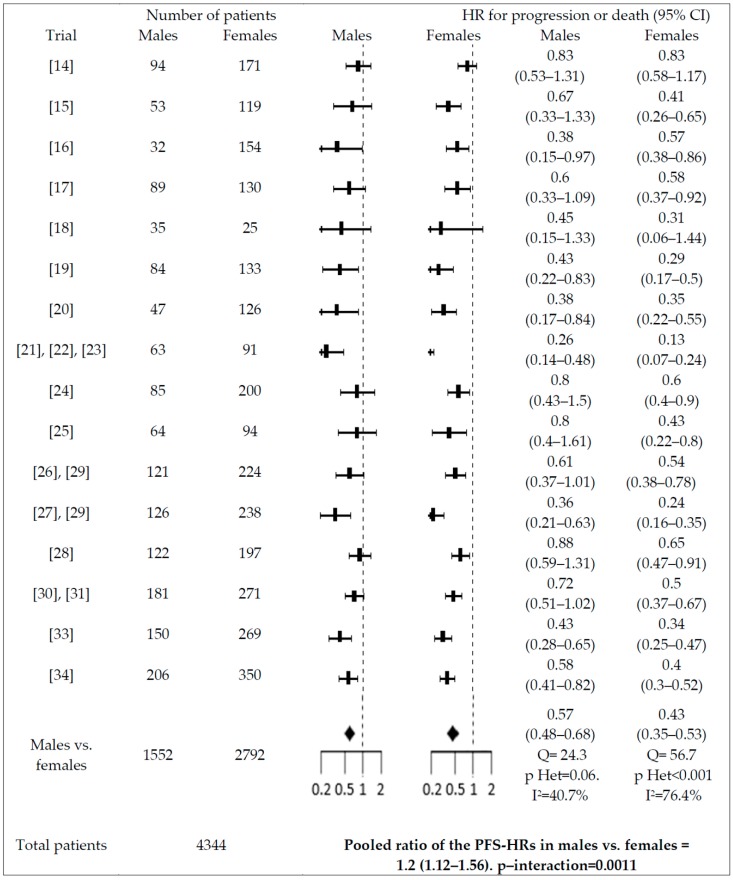
Interaction between PFS-HR and sex. PFS-HRs are separately reported for males vs. females. Pooled PFS-HRs in males and females and pooled ratio of the PFS-HRs in males vs. females are reported at the bottom of the figure.

**Figure 4:**
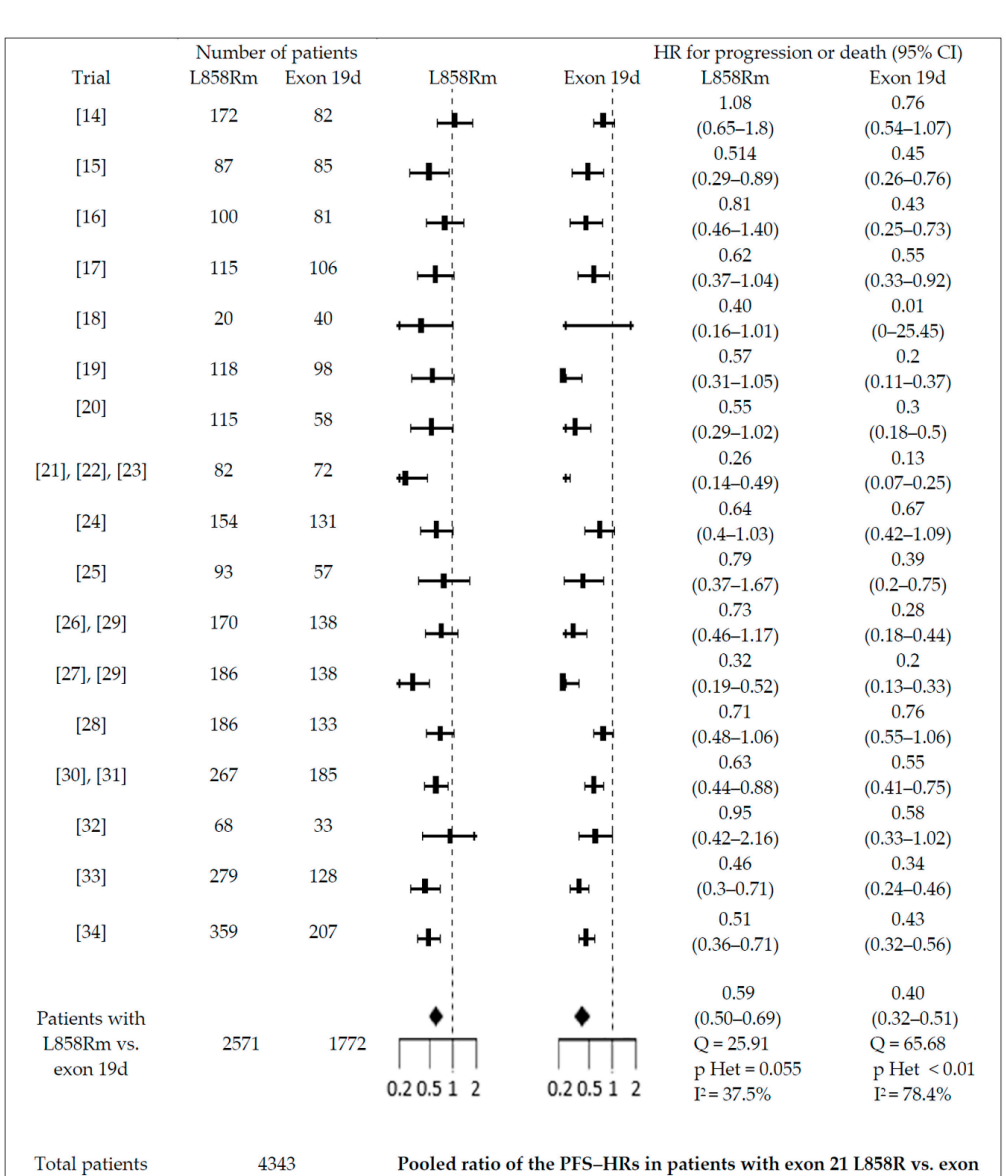
Interaction between PFS-HR and EGFR mutation. PFS-HRs are separately reported for patients with exon 21 L858R mutation vs. exon 19 deletion. Pooled PFS-HRs in patients with exon 21 L858R mutation vs. exon 19 deletion and pooled ratio of the PFS-HRs in patients with exon 21 L858R mutation vs. exon 19 deletion are reported at the bottom of the figure. m = mutation; d = deletion.

**Figure 5:**
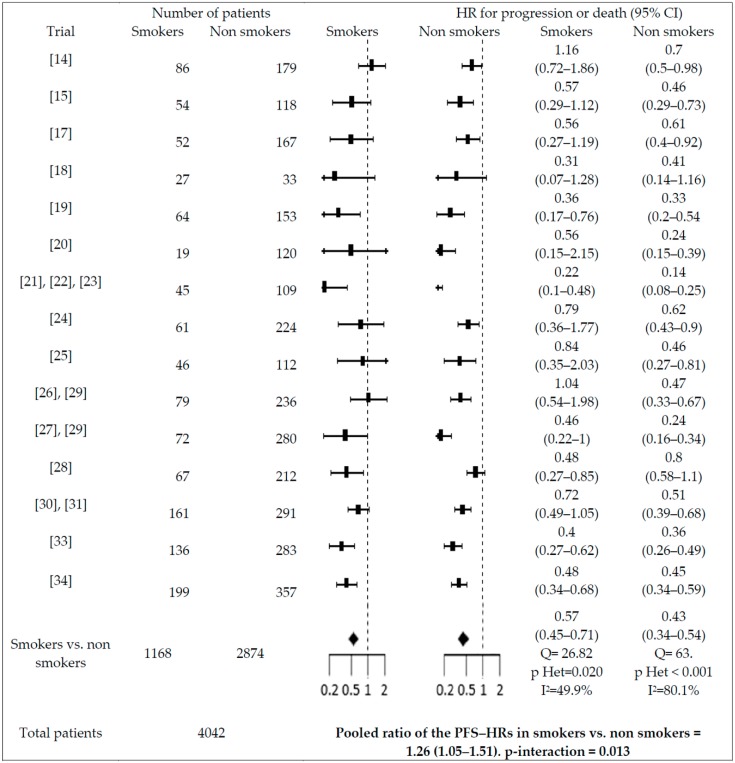
Interactions between PFS-HR and smoking history. PFS-HRs are separately reported for smokers vs. non-smokers. Pooled PFS-HRs in smokers vs. non-smokers and pooled ratio of the PFS-HRs in smokers vs. non-smokers are reported at the bottom of the figure.

Significant interactions with PFS were found for gender (males vs. females; pooled ratio of the PFS-HRs = 1.2, 95% CI = 1.12–1.56), smoking history (smokers vs. non-smokers; pooled ratio of the PFS-HRs = 1.26; 95% CI = 1.05–1.51), and type of EGFR mutation (patients with exon 21 L858 mutation vs. exon 19 deletion; pooled ratio of the PFS-HRs = 1.39; 95% CI = 1.18–1.63)(see Figure 3, Figure 4 and Figure 5). No significant interaction (*p* > 0.05) was reported between PFS-HR and performance status (PS = 0 vs. 1; pooled ratio of the PFS-HRs = 0.9; 95% CI = 0.72–1.12; *p* = 0.33; 5 trials included), age (<65 vs. ≥65 years old; pooled ratio of the PFS-HRs = 0.93; 95% CI 0.77–1.13; 11 trials included), ethnicity (Asian vs. non-Asian; pooled ratio of the PFS-HRs = 0.89; 95% CI 0.64–1.23; 6 trials included), brain metastases (absence vs. presence; pooled ratio of the PFS-HRs = 1.03; 95% CI 0.79–1.15; 6 trials included).

As regards OS, no significant interaction (*p* interaction > 0.05) was found with sex (males vs. females; pooled ratio of the OS-HRs = 0.96; 95% CI 0.74–1.25), smoking history (smokers vs. non-smokers; pooled ratio of the OS-HRs = 1.02; 95% CI 0.77–1.37), type of EGFR mutation (patients with exon 21 mutation vs. exon 19 mutation; pooled ratio of the OS-HRs = 1.25; 95% CI 0.86–1.82), age (<65 vs. 65 years old; pooled ratio of the OS-HRs = 0.97; 95% CI 0.70–1.35; 4 trials included); PS (PS = 0 vs. 1; pooled ratio of the OS-HRs = 1.29; 95% CI = 0.89–1.87; 3 trials included); ethnicity(Asian vs. non-Asian; pooled ratio of the OS-HRs = 0.97; 95% CI 0.70–1.35; 2 trials included).

**Figure 6:**
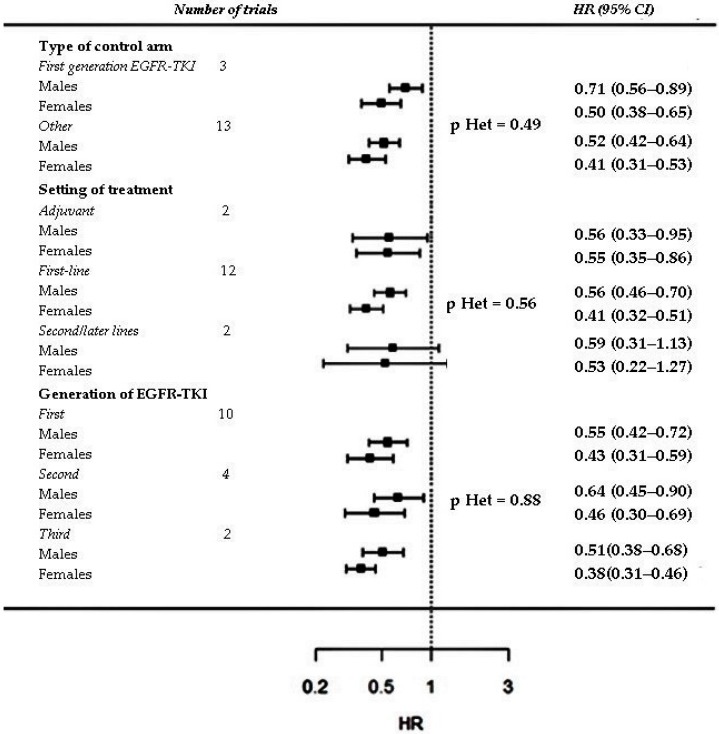
Subgroup analysis of PFS interaction with sex. Trials are grouped according to type of control arm (first-generation EGFR-TKI vs. other), setting/line of treatment (adjuvant vs. first line vs. second/later lines) and generation of the EGFR-TKI tested in the experimental arm (first vs. second vs. third). P value for heterogeneity (p Het) among pooled ratios of the PFS-HRs in males vs. females estimated in trial subgroups is provided.

**Figure 7:**
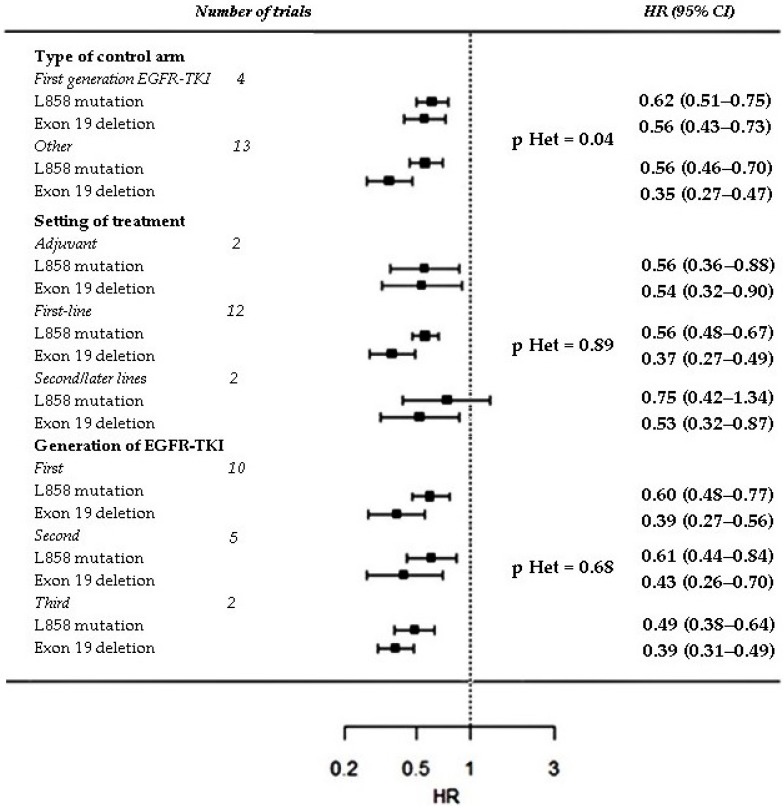
Subgroup analysis of PFS interaction with type of EGFR mutation. Trials are grouped according to type of control arm (first-generation EGFR-TKI vs. other), setting/line of treatment (adjuvant vs. first line vs. other) and generation of the EGFR-TKI tested in the experimental arm (first vs. second vs. third). P value for heterogeneity (p Het) among pooled ratios of the PFS-HRs in patients with exon 21 L858R mutation vs. exon 19 deletion estimated in trial subgroups is provided.

**Figure 8:**
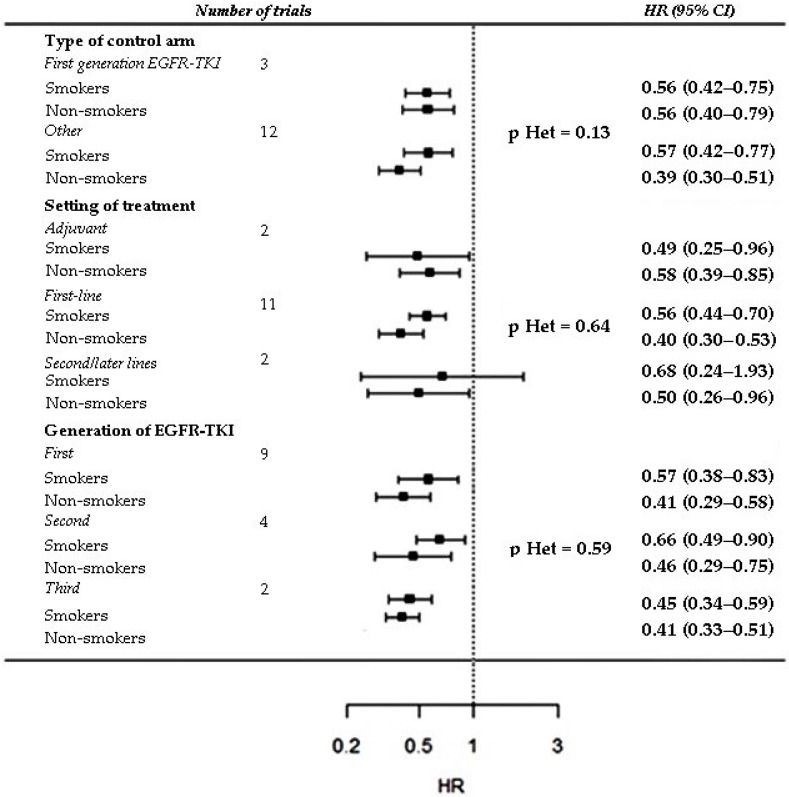
Subgroup analysis of PFS interaction with smoking status. Trials are grouped according to type of control arm (first-generation EGFR-TKI vs. other), setting/line of treatment (adjuvant vs. first line vs. other) and generation of the EGFR-TKI tested in the experimental arm (first vs. second vs. third). *p* value for heterogeneity (*p* Het) among pooled ratios of the PFS-HRs in smokers vs. non-smokers computed in trial subgroups is provided.

Subgroup analysis based on type of control arm, setting and generation of the EGFR-TKI was performed only for variables for which a significant interaction was reported: sex, smoking history and type of EGFR mutation. No significant heterogeneity was found among the pooled ratios of the PFS-HRs in males vs. females, in smokers vs. non smokers and in patients with exon 21 mutation vs. exon 19 deletion computed in the trial sub-groups, except for a significant heterogeneity among the pooled ratios of the PFS-HRs in patients with exon 21 mutation vs. exon 19 mutation computed in trials grouped according type of control arm (*p* = 0.0462) (Figure 6, Figure 7 and Figure 8).

## 3. Discussion

Gender is capable of influencing cancer prognosis, as well as response and adverse events associated with systemic anticancer therapy on the basis of profound biological differences between males and females with regard to genetic polymorphisms of drug metabolizing enzymes [35], sex hormone levels [36], as well as in the immune system [37]. In patients with NSCLC, gender has an established independent prognostic value. In a large population-based study [38] involving 11,678 Taiwanese patients with lung adenocarcinoma diagnosed from 2011 to 2014 (5189 males and 6489 females), female gender had a similar favorable impact on survival both in EGFR-mutated patients (HR: 0.86 (95% CI, 0.80–0.93); *p* < 0.001) and in EGFR-wild type patients (HR = 0.88; 95% CI, 0.81–0.96; *p* = 0.004). In this study, smoking was also associated with increased risk of death both in EGFR-mutant and -wild type patients (HR: 1.20; 95% CI, 1.10–1.30; and HR: 1.33; 95% CI, 1.23–1.47, respectively; both *p* < 0.001). Whether such factors may affect EGFR-TKI efficacy, apart from prognosis, is a subject of investigation. One meta-analysis including individual data from 1231 patients enrolled in 6 RTCs of EGFR-TKI vs. chemotherapy in EGFR-mutant NSCLC patients did not find any differences in pooled OS-HRs in women (HR = 1.02; 95% CI = 0.86–1.21) and men (HR = 0.98; 95% CI = 0.76–1.27). Although different pooled PFS-HRs were reported in women (HR = 0.34; 95% CI = 0.29–0.41) vs. in men (HR = 0.42; 95% CI = 0.33–0.54), such a numerical difference was not associated with any statistically meaningful heterogeneity according to the authors [12]. Similar results were obtained in the meta-analysis by Hasegawa et al. that included published data of 1649 EGFR-mutant NSCLC patients enrolled in 7 prospective randomized trials. In this meta-analysis, pooled PFS-HRs for women and men were 0.31 (95% CI: 0.23–0.40) and 0.43 (95% CI: 0.32–0.57), but meta-regression analysis of the HRs was not reported as statistically significant (*p* = 0.090) [11]. Finally, in the meta-analysis by Pinto et al. [10] that included 1425 EGFR-mutant NSCLC patients receiving EGFR-TKIs in RCTs, the pooled PFS-HR was 10% higher in males vs. females, but the statistical significance of this numerical difference was not formally analyzed by the use of an appropriate statistical test.

Our meta-analysis is the first to provide statistically robust evidence supporting gender-based heterogeneity in the risk of progression or death with the use of first-, second-, and third-generation EGFR-TKIs in EGFR-mutant patients enrolled in RCTs. In order to strengthen the quality of our analysis, we only included data obtained in EGFR-mutant populations treated with EGFR-TKIs. A different approach was followed by Wang et al. [39], who included clinical trials testing different agents and, among these, clinical trials of EGFR-TKIs in NSCLC patients unselected for EGFR status, which could interact with gender and act as a confounder. Furthermore, we did subgroup analysis that showed that interactions of HR-PFS with gender were not affected by line of treatment, type of comparator, or generation of the EGFR-TKI used. Apart from gender, type of mutation and smoking status were also associated with PFS-HR, but not with OS-HR, while no interaction was found for performance status, ethnicity, age, as well as presence of brain metastases. With regards to smoking habit and type of EGFR mutation, our results are consistent with those reported in other published meta-analyses [11], although our meta-analysis has the merit of including a larger sample size including patients receiving third-generation EGFR-TKIs. As an example, the meta-analysis of published data done by Hasegawa et al. [11] showed that the pooled HR-PFS was 0.29 (95% confidence interval [CI]: 0.21–0.39) for never-smokers and 0.54 (95% CI: 0.38–0.76) for ever-smokers (*p* < 0.007 by meta-regression), while the pooled PFS HR for exon 19 mutation was 0.25 (95% CI: 0.19–0.31) and 0.44 for exon 21 substitution (95% CI: 0.34–0.57) (*p* < 0.001 by meta-regression analysis). In subgroup analysis, we noted that smoking and type of EGFR mutation may only marginally affect the outcome of third- and second-generation EGFR-TKIs vs. first-generation EGFR-TKIs. In fact, when a first-generation EGFR-TKI was used as a control in RCTs of second-and third-generation EGFR-TKIs, pooled PFS-HRs for exon 21 mutation vs. exon 19 deletion were 0.62 (0.51–0.75) vs. 0.56 (0.43–0.73), respectively, while pooled PFS-HRs for smokers vs. non-smokers were 0.56 (0.42–0.75) and 0.56 (0.40–0.79), respectively, with a *P*-value for heterogeneity significant or borderline significant. Also, we noted that in all of the three less vs. more favorable groups (male sex, exon 21 mutation, smokers), a lower heterogeneity of EGFR-TKI was reported (I < 50%), which could at least partially be explained by the significantly lower efficacy, which decreases the variability of the results, as reported also by Conforti et al. [40]. Finally, it is important to note that the pooled patient population was sufficiently large to show that patients on EGFR-TKI also had better survival vs. controls (OS-HR = 0.88; 95% CI 0.79–0.98), while previous meta-analyses had failed to do so [12,41].

The strengths of our meta-analysis lie in the robust statistical methods used, as well as in the selection of homogenous populations of NSCLC EGFR-mutant patients receiving EGFR-TKI vs. other interventions in RCTs. Although we analyzed findings from >4000 patients and obtained a pooled sample as large as never analyzed before, to the best of our knowledge, the lack of individual data must be acknowledged as a limitation. Furthermore, while categories identified by gender, ECOG PS, presence of brain metastases, type of EGFR mutation, age, and smoking habit are well-defined, we are aware that the classification of patients as Asian and non-Asian may be too imprecise to capture any effect of ethnicity. In fact, although intragroup differences in pharmacogenetics, acquired mutation patterns, and environmental conditions are expected among both Asian and non-Asian individuals, our analysis was limited by the lack of more detailed data regarding ethnicity. Finally, we were unable to explore whether sex, type of mutation, and smoking habits may independently affect EGFR-TKI efficacy. If so, male smokers with exon 21 mutations may derive limited benefit from single-agent EGFR-TKIs and represent a subgroup of patients that may benefit from alternative approaches (e.g., combination of EGFR-TKI + immunotherapy [42]).

## 4. Methods

### 4.1. Search Strategy

The search for relevant articles was conducted by querying PubMed/MEDLINE and Embase, according to the PRISMA guidelines. Articles published since inception until 31 May 2019 were evaluated for inclusion in the systematic review. Abstracts and presentations from ASCO (American Society of Clinical Oncology) and ESMO (European Society of Medical Oncology) from 2010 until 2019 were also reviewed. 

The search terms included the following keywords: “EGFR-TKI”, “epidermal growth factor receptor-tyrosine kinase inhibitor”, “gefitinib”, “erlotinib”, “icotinib”, “dacomitinib”, “osimertinib”, “afatinib”.

We also reviewed the references of articles finally included in this meta-analysis. When duplicate publications were found, the most updated data were considered.

We included all articles reporting about phase II and III randomized control clinical trials conducted in patients with non-small cell lung cancer randomized to EGFR-TKI vs. any other treatment. Eligible articles were required to report subgroup analysis in EGFR-mutated patients of progression-free survival (PFS) (or disease-free survival (DFS) in trials conducted in the adjuvant setting) and/or overall survival (OS) by at least one of the following seven variables categorized as specified here: gender (male vs. female), age (<65 vs. ≥65 years old), ethnicity (Asian vs. non-Asian), smoking habit (current vs. never smoker), brain metastasis (presence vs. absence), type of EGFR mutation (exon 19 mutation vs. L858R mutation), ECOG PS (0 vs. 1). We also included trials comparing third-generation (osimertinib) and second-generation (dacomitinib, afatinib) EGFR-TKIs vs. first-generation EGFR-TKIs (erlotinib, gefitinib, icotinib).

Subgroups of RCTs were identified by considering the EGFR-TKI generation of the interventional arm (first vs. second vs. third EGFR-TKI generation), type of control arm (first-generation EGFR-TKI vs. other), setting (adjuvant vs. first line vs. second/later lines of therapy).

### 4.2. Data Analysis

The primary objective of the meta-analysis was to explore the influence of seven commonly available baseline clinical, demographic or genetic variables (gender, age, ethnicity, smoking habit, ECOG PS, brain metastasis, EGFR mutation) on PFS-HR and/or OS-HR (efficacy outcomes) reported in RCTs of EGFR-TKIs in NSCLC patients with activating EGFR mutations. PFS-HRs and OS-HRs for each modality of the predictor variables were reported for each trial. We evaluated heterogeneity among studies using the χ^2^ Q test and I^2^ statistics. For the Q test, significant heterogeneity was declared if *p* < 0.05, while I^2^ values > 50% were considered to indicate evident heterogeneity. Pooled PFS-HR and pooled OS-HR were calculated using random-effects models. The pooled ratio of the PFS-HRs and OS-HRs were reported together with their corresponding 95% CI. Results were also graphically displayed as a forest plot.

To explore whether the predictor variables may influence PFS and OS, an interaction test was performed following the approach reported by Fisher et al. [13]. This approach avoids the risk of ecological bias in testing heterogeneity among groups, by computing within-trial interaction as the ratio of the reported HRs in the two groups, and then these trial-specific interaction HRs are pooled across trials using a random-effects model.

The secondary objective of the meta-analysis was to explore whether the interactions associated with the primary objective were influenced by setting, EGFR-TKI generation, and type of comparator arm. These three variables identifying subgroups of RCTs as specified above were included in the model as a moderator to test if some heterogeneity among the trial-specific interaction HRs may be due to their influence. 

Reporting bias was evaluated by assessing visual asymmetry on funnel plots of global HRs against standard errors. To examine whether the association between effect sizes and the related standard errors was greater than expected to occur by chance, the regression test for funnel plot asymmetry was carried out. Since tests for funnel plot asymmetry typically have low power, results must be interpreted with caution.

As regards PFS-HR, funnel plots and corresponding regression tests were also performed considering setting, EGFR-TKI generation, and type of comparator arm as moderator variable. For models involving moderators, the residuals were analyzed instead of effect sizes. The statistical software R version 3.2.5 (13) was used for all statistical analyses. Meta-analysis was performed using metafor package, version 2.1–0, with *p* < 0.05 considered as statistically meaningful. Prof. Dolce, a biostatistician at the Department of Public Health of University Federico II of Naples, was responsible for the statistical analysis, which was internally reviewed for accuracy by a senior biostatistician of the Department.

## 5. Conclusions

Our meta-analysis of RCTs of first-, second- and third-generation EGFR-TKIs identified sex, type of EGFR mutation, and smoking status as predictor variables of treatment efficacy. These findings should be interpreted with caution as regards their implications for clinical practice, but may provide valuable insights for the design of clinical trials exploring novel treatment options for selected EGFR-mutant NSCLC patients.

## Figures and Tables

**Table 1:** Main characteristics of included trials. No = number; pts = patients; HR = Hazard Ratio; CI = Confidence Interval; WBI = Whole Brain Irradiation.

Trial	Phase	Setting (0 = Adjuvant; 1 = First Line; 2 = Second or Subsequent Lines)	Jadad Score (0–5)	No. Evaluable pts	Age in Years (Range)	Median Follow-up in Months	Intervention	Control	HR for Progression—95% CI	HR for Death—95% CI		
[14]	3	2	5	265	59 (33–79)		GEFITINIB + CISPLATIN + PEMETREXED	PLACEBO + CISPLATIN + PEMETREXED	0.86 (0.65–1.13)			
[15]	3	1	3	172	64 (34–75)	2.7	GEFITINIB	CISPLATIN + DOCETAXEL	0.48 (0.33–0.71)	1.63 (0.75–3.58)		
[16]	3	1	3	186			GEFITINIB	CARBOPLATIN + PACLITAXEL	0.54 (0.38–0.79)			
[17]	3	0	3	219	59 (26–76)	36.5	GEFITINIB	VINORELBINE + CISPLATIN	0.58 (0.4–0.83)			
[18]	2	0	3	60	57 (32–78)	30.6	GEFITINIB	PEMETREXED + CARBOPLATIN	0.37 (0.16–0.85)			
[19]	3	1	3	217	56 (30–79)	28.9/27.1	ERLOTINIB	GEMCITABINE + CISPLATIN	0.42 (0.27–0.66)	0.91 (0.63–1.31)		
[20]	3	1	3	173	65 (24–72)	18.9/14.4	ERLOTINIB	PLATINUM + DOCETAXEL/GEMCITABINE	0.37 (0.25–0.54)	1.04 (0.65–1.68)		
[21,22,23]	3	1	3	154	54 (31–78)		ERLOTINIB	GEMCITABINE + CARBOPLATIN	0.16 (0.11–0.26)	1.19 (0.83–1.71)		
[24]	3	1	3	285	56 (30–76)	18	ICOTINIB	CISPLATIN + PEMETREXED	0.65 (0.46–0.91)	0.97 (0.72–1.31)		
[25]	3	1	3	158	58 (48–64)	16.5	ICOTINIB	WBI	0.56 (0.36–0.9)	0.93 (0.6–1.44)		
[26,29]	3	1	3	345	61 (28–86)	16.4	AFATINIB	CISPLATIN + PEMETREXED	0.58 (0.43–0.78)	0.78 (0.58–1.06)		
[27,29]	3	1	3	364	58 (49–65)	16	AFATINIB	GEMCITABINE + CISPLATIN	0.28 (0.2–0.39)	0.83 (0.63–1.09)		
[28]	2	1	3	319	63 (30–89)		AFATINIB	GEFITINIB	0.73 (0.57–0.95)			
[30,31]	3	1	3	452	61 (53–68)	22.1 (PFS) 31.3 (OS)	DACOMITINIB	GEFITINIB	0.58 (0.4–0.73)	0.80 (0.61–1.04)		
[32] ARCHER 1009	3	2	5	121	61 (32–84)		DACOMITINIB	ERLOTINIB	0.71 (0.45–1.12)	0.95 (0.59–1.53)		
[32] A7471028	2	2	3		62 (34–79)							
[33]	3	2	3	419	62 (20–90)	8.3	OSIMERTINIB	PLATINUM + PEMETREXED	0.3 (0.23–0.41)			
[34]	3	1	5	556	64 (26–93)	15	OSIMERTINIB	GEFITINIB OR ERLOTINIB	0.46 (0.37–0.57)	0.63 (0.45–0.88)		
